# The Role of Empathy and Life Satisfaction in Internet and Smartphone Use Disorder

**DOI:** 10.3389/fpsyg.2018.00398

**Published:** 2018-03-27

**Authors:** Bernd Lachmann, Cornelia Sindermann, Rayna Y. Sariyska, Ruixue Luo, Martin C. Melchers, Benjamin Becker, Andrew J. Cooper, Christian Montag

**Affiliations:** ^1^Institute of Psychology and Education, Ulm University, Ulm, Germany; ^2^The Clinical Hospital of Chengdu Brain Science Institute, MOE Key Lab for Neuroinformation, University of Electronic Science and Technology of China, Chengdu, China; ^3^Department of Psychology, University of Bonn, Bonn, Germany; ^4^Department of Psychology, Goldsmiths, University of London, London, United Kingdom

**Keywords:** problematic internet use, problematic smartphone use, life satisfaction, cross-cultural, personal distress

## Abstract

Recent studies have yielded initial evidence for an association between Internet Use Disorder (IUD), empathy, and life satisfaction. In the present study we sought to replicate these previous findings, and then to extend this research by also examining the relationship between empathy, life satisfaction, and the related phenomenon of Smartphone Use Disorder (SUD). The present study included independent samples from China (*N* = 612, 162 females) and Germany (*N* = 304, 207 females), with the same set of questionnaires administered to both samples. IUD was measured with Pawlikowski's s-IAT and SUD was assessed with the short version of Kwon's Smartphone Addiction Scale. The Interpersonal Reactivity Index (IRI) was used to assess individual differences in empathy. Please note that for the German sample data on the empathy quotient (EQ) are also available. Life satisfaction data were collected using items from the SOEP-Questionnaire (Socio-Economic Panel, Germany). In both of our samples we replicated previous findings showing the association between higher IUD, lower empathy, and lower life satisfaction scores. In addition, individuals with higher SUD showed higher scores on the IRI Personal Distress scale in China and Germany, while further associations between IRI dimensions and SUD were only found in the Chinese sample. Personal Distress is known to be highly correlated with the personality trait of Neuroticism, hence higher stress/negative emotionality in tense social situations is related to SUD. In the present study we confirm earlier findings showing the relationship between empathy, life satisfaction, and IUD, and extend some of these findings to SUD. We also emphasize the importance of cross-cultural studies when investigating IUD/SUD in the context of empathy and life satisfaction.

## Introduction

The development and use of digital devices has changed the way people communicate, gather information, and access the Internet (e.g., Cui, 2016; Hayes et al., 2016). Above all, one device—the smartphone—has the potential to greatly influence human behavior due to its specific characteristics and functions: it is small enough to accompany the user in nearly all situations, it offers a variety of different functions, and it is particularly user friendly (e.g., Poushter, 2016; Wang et al., 2016). These characteristics mean the smartphone has the potential to integrate very closely and conveniently with an individual's daily life. The proximity and convenience of the smartphone, however, enhances the risk of over-use of the device, which over time could lead to addictive behavior (Lee et al., 2014; Duke and Montag, 2017a; Jo et al., 2018). Evidence for negative consequences of excessive use has been primarily reported in populations from Asian countries where a strong affinity to the smartphone developed years earlier than in, for example, European countries (Lee et al., 2016; Lee and Lee, 2017; Park and Choi, 2017). Furthermore, the existing literature suggests that adolescents in particular are at elevated risk for developing problematic and addictive behaviors related to smartphone use, and these behaviors have been associated with negative consequences for everyday functioning and mental health (Bae, 2017; Duke and Montag, 2017b; Lee et al., 2017). Consequently, one of the first questionnaires that assess individual levels of excessive use and addictive behaviors in relation to the smartphone was developed in Asia: the smartphone addiction scale (SAS) (Kwon et al., 2013). Despite this, the majority of users do not indicate problematic behavior in relation to their smartphone, and to date little is known about factors associated with an increased risk of developing a smartphone use disorder (SUD).

Smartphone use is strongly linked to the Internet – Internet access is a prerequisite for many functions of the smartphone (Choi et al., 2015; Montag et al., 2015a,b), particularly those with stronger addictive potential, such as communication with social networks (Montag et al., 2017). Of course, the Internet can also be accessed via other devices, including laptops or tablets, but since smartphone use and the Internet are interwoven to such a close extent, it seems plausible that the development of SUD should have its roots, at least partially, in the development of Internet use disorder (IUD) (Duke and Montag, 2017a; Lachmann et al., 2017a). Initial empirical support for this notion comes from previous research reporting associations between IUD and SUD as high as *r* = 0.65 (e.g., Ha et al., 2008). Lower, but still substantial, correlations have also been observed in a study from Germany (*rho* = 0.53 in Lachmann et al., 2017a). However, despite the high co-occurrence of the disorders, it remains unclear whether both phenomena are driven by the same factors.

Given that IUD is increasingly recognized as an emerging health issue (Kuss and Lopez-Fernandez, 2016), a growing number of studies have aimed to determine the factors that underlie the development of IUD. Examples of this research include outlining specific personality profiles that allow a better characterization of persons with IUD (Shek et al., 2008), the development of clear and coherent diagnostic criteria (Tao et al., 2010), the development of a number of models to explain IUD (Davis, 2001; Caplan, 2010; Brand et al., 2016), and establishing associations between IUD and pathological factors like depression or anxiety (Dalbudak et al., 2013; Ostovar et al., 2016). In this context, two strategies in particular have received increasing attention during recent years: examining associations between personality factors and IUD (for an overview: Floros and Siomos, 2014; Montag and Reuter, 2015), and determining patterns of co-morbidity between psychiatric disorders and IUD (for an overview: Carli et al., 2013; Kuss et al., 2014). While the latter associations are particularly important in determining the current phase of a disorder, the investigation of personality could lead to the identification of vulnerability factors linked to the development of IUD and to help identify individuals at an increased risk of developing IUD. This relation is also outlined in a new model explaining the development and maintenance of IUD, the I-PACE (Interaction of Person-Affect-Cognition-Execution) model from Brand et al. (2016). Aside from factors such as the biopsychological constitution of a person (e.g., genetics), motives for using the Internet, psychopathological factors, or social cognitions, personality factors are considered to play a crucial role when developing or maintaining IUD (Brand, 2017). Previous studies in this regard suggest that, for example, low self-directedness (Montag et al., 2010, 2011; Hahn et al., 2017), low self-esteem (Sariyska et al., 2014), and low conscientiousness (Stavropoulos et al., 2016) might be predisposing factors that render individuals vulnerable for the development of IUD. Furthermore, also associations between lower life satisfaction and higher IUD (Shahnaz and Karim, 2014; Pontes et al., 2015) as well as lower empathy levels and higher IUD (Melchers et al., 2015; Jing et al., 2017) have been observed. Until now, the research on the association between empathy and life satisfaction and IUD/SUD is very limited. It should be noted here that there is evidence showing a positive association between life satisfaction and Internet use, but only in terms of Internet adoption and not IUD (e.g., Lissitsa and Chachashvili-Bolotin, 2016). However, in general the link between life satisfaction and IUD is negative as demonstrated by a meta-analysis (Cheng and Li, 2014) and the majority of the conducted studies in this field. But clearly, associations might be positive when the Internet is used in a “healthy” way. Within the framework of dimensional models of psychological disorders, the approach targeting pre-clinical levels of IUD might facilitate identifying individuals at increased risk before the development of a clinically relevant IUD and thus support the development of preventive strategies.

**Figure 1:**
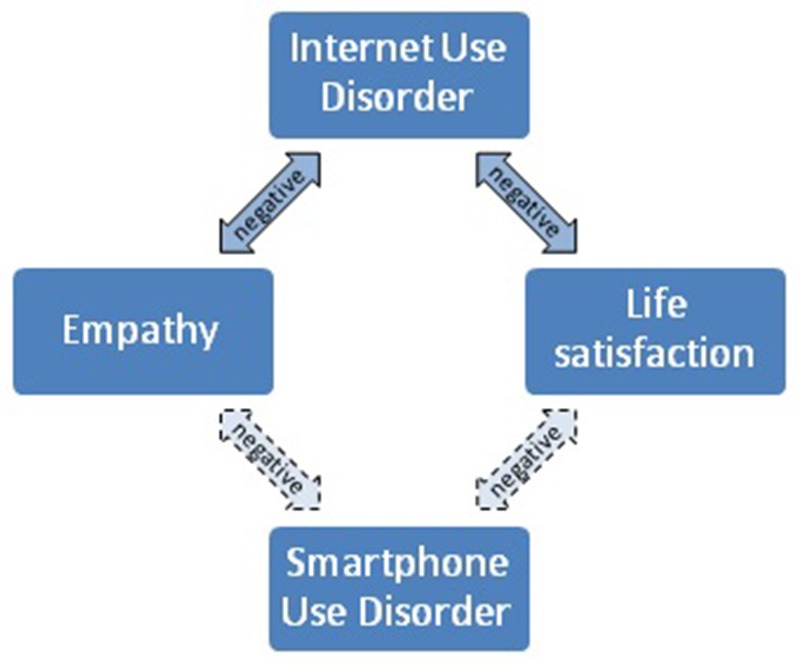
Findings from the literature (solid arrows) and presumed (dashed arrows) associations (to be investigated in the present study) between IUD, empathy, life satisfaction, and SUD.

Of particular interest in this context are converging findings from two recent studies reporting associations between lower empathy and higher levels of IUD (Melchers et al., 2015; Jing et al., 2017). Empathy can be defined as a trait characterizing, firstly, an individual's ability to correctly understand another person's emotions, thoughts, feelings, and motives (cognitive empathy), and, secondly, an individual's ability to respond to the emotional state of others with appropriate affective reactions (affective empathy, Baron-Cohen and Wheelwright, 2004). Together, these two aspects play an important role for effective social interactions. This is supported by the fact that empathy has been robustly linked to agreeableness, one of the factors of the Big Five Model of Personality (Melchers et al., 2016). Of note, empathy has also been positively related to life satisfaction (e.g., Bourgault et al., 2015; Choi et al., 2016; Caro et al., 2017), and lower life satisfaction has been associated with higher levels of IUD (e.g., Shahnaz and Karim, 2014; Lachmann et al., 2016; Longstreet and Brooks, 2017). The association reported in the literature between low empathy and high IUD is plausible since high levels of Internet use could be behavior that compensates for low levels of social well-being and competency. Considering both the overlap between IUD and SUD (e.g., Ha et al., 2008; Kwon et al., 2013) and the recent findings suggesting common personality dimensions predicting both IUD and SUD (Lachmann et al., 2017a), then the question that arises is the extent to which lower empathy and life satisfaction are linked with increased levels of SUD. For a visualization of these relationships, please refer to Figure 1.

As outlined above, most previous research on vulnerability factors for IUD/SUD has been conducted in Asian populations (Block, 2008). Adding to this, there are several findings reporting differences in how the Internet is used depending on the specific cultural predisposition. For example, one study suggested that Chinese students spend much more time online on independent academic activities than their counterparts from the USA (Shostya, 2015). Other studies have observed differences between Chinese and American/British students in how the Internet was used in school and at home (Li and Kirkup, 2007; Lei et al., 2009). Furthermore, also differences in the levels of empathy in China and Europe (Melchers et al., 2015) have been found (the levels of empathy in Europe were higher, but this needs to be further investigated in future studies) as well as cross-cultural influence on life satisfaction (Schimmack et al., 2002; Lachmann et al., 2018). Against this background, examining potential cultural differences in the proposed associations between IUD/SUD, empathy, and life satisfaction appears particularly relevant. To this end, the present study included data from samples in China and Germany.

In summary, the present study aimed to extend the previous research showing a negative association between empathy and IUD by examining the relationship between empathy and SUD. Similarly, given the previous research showing a negative association between life satisfaction and IUD, the present study also sought to examine the relationship between life satisfaction and SUD. These relationships were examined across samples from two distinct cultures to highlight whether these specified relationships held across cultural context, or whether the pattern of relationships varied across culture.

## Materials and methods

### Participants

Data were collected from two samples for this study. Data for the first sample were collected at the University of Electronic Science and Technology of China, Chengdu, China (*N* = 612; 162 females). The mean age of this sample was 21.55 (*SD* = 2.44), ranging from 18 years to 32 years. The level of education of the participants was distributed as follows: 59.1% held a Baccalaureate-Diploma and 40.9% had a university degree. The second sample was collected in Germany (*N* = 304; 207 females) and had a mean age of 24.05 (*SD* = 8.85), ranging from 18 years to 63 years. Most, but not all, of the participants were recruited from Ulm University, Germany. The remaining participants were adults recruited from the general community in Germany. Overall, 0.1% of the participants had no school leaving certificate, 6.9% had a secondary school leaving certificate, 70.2% held a Baccalaureate-Diploma, and 22.8% had a university degree. Participation was voluntary in both samples. A digital consent form had to be signed prior to taking part in the current study. As an incentive for participation, participants could request a short report concerning the findings of the study. The study was approved by the local ethics committees at Ulm University, Germany, and the University of Electronic Science and Technology of China, Chengdu, China.

### Materials

The following German or Chinese version questionnaires were used to collect data from both samples.

Empathy was measured using the Interpersonal Reactivity Index (IRI; Davis, 1980). The IRI consists of 28 items which are answered on a 5-point Likert scale ranging from “Does not describe me well” to “Describes me well.” According to Davis (1980), four subscales can be utilized: “Perspective Taking” (PT), which is the tendency to spontaneously adopt the psychological point of view of others; “Fantasy” (FS), which is the ability to transpose oneself imaginatively into the feelings and actions of fictitious characters in books, movies, and plays; “Empathic Concern” (EC) which assesses “other-oriented” feelings of sympathy and concern for unfortunate others; and “Personal Distress” (PD) which measures “self-oriented” feelings of personal anxiety and unease in tense interpersonal settings. The following Cronbach's α were found in the Chinese sample: PT (α = 0.65), FS (α = 0.65), EC (α = 0.66), and PD (α = 0.67). For the German sample, Cronbach's α were: PT (α = 0.78), FS (α = 0.82), EC (α = 0.84), and PD (α = 0.76).

Data on IUD were collected using the short Internet addiction test (s-IAT) from Pawlikowski et al. (2013). In contrast to the Internet addiction test (Young, 1998) which has 20 items, the s-IAT consists of only 12 items. Answers are provided using a 5-point Likert scale ranging from “Never” to “Very often.” The proposed cut-off values from Pawlikowski et al. (2013) were used to distinguish between normal use (score < 31), moderate use (score: 31–37), and problematic use (score > 37). The s-IAT has been administered in several studies (e.g., Montag et al., 2015b; Lachmann et al., 2017b; Sariyska et al., 2017) yielding good internal consistencies. Cronbach's α in the Chinese sample was α = 0.88 and in the German sample was α = 0.86.

A short version of the SAS was used to gather data on SUD (Kwon et al., 2013). The questionnaire consists of 10 items which are scored on a 6-point Likert scale ranging from “Strongly disagree” to “Strongly agree.” To discriminate between normal and pathologic use of smartphones, gender-specific cut-off values (female = 33; male = 31) were used, as suggested by Kwon et al. (2013). Cronbach's α in the Chinese sample was α = 0.87 and in the German sample was α = 0.79.

To assess life satisfaction, we asked seven questions gathered from the German Socio-Economic Panel (SOEP) (Siedler et al., 2008). The following areas of life satisfaction were targeted: health, job, income, housing, leisure, family, and overall life satisfaction. As recommended, the item referring to overall life satisfaction (“How satisfied are you with your life overall?”) was asked at the end of the questionnaire to avoid possible interference with the items referring to specific areas of life satisfaction. It should be noted that while all items from this questionnaire are considered to be distinct, they do also overlap to a certain degree. Also, it is important to note that overall life satisfaction is not scored by simply summing all specific life satisfaction variables (to get more detailed information concerning life satisfaction and the relationships between life satisfaction variables, please refer to Rojas, 2006; Erdogan et al., 2012; Lachmann et al., 2018). The items for this measure were answered using a Likert scale ranging from 0 (“Completely dissatisfied”) to 10 (“Completely satisfied”).

### Procedure

In both countries the collection of data was conducted via an online platform. Promotion of the study occurred in lectures, via bulletin boards, with the help of flyers, and via online social networks. Participants had to provide an e-mail address to get an invitation with a link to the questionnaires. By hitting the “send” button at the end of the questionnaire, all data were transferred to our server and no further changes to responses were possible.

### Statistical analysis

Differences in gender and age across sample were tested. Gender differences concerning the questionnaire responses were analyzed using *t*-tests. Correlations between age, IUD/SUD, empathy, and life satisfaction were examined to test the hypothesized associations (Figure 1). Cultural differences were examined by comparing the correlations in the samples from China and Germany by means of Fisher's r to z transformation. To get information concerning the level of IUD/SUD in both samples, the suggested cut-off values described above (Kwon et al., 2013; Pawlikowski et al., 2013) were used. All analyses were Bonferroni corrected to control for multiple testing. The analyses were conducted using the SPSS version 22.0 for Windows (IBM SPSS Statistics, Chicago, IL, USA).

## Results

Inspection of the data revealed no missing data or outliers, and a normal distribution for all variables. For the descriptive statistics for the IUD/SUD, empathy, and life satisfaction variables, please refer to Table 1. Age and gender differed significantly across the Chinese and German samples. Participants in the Chinese sample were younger than in the German sample [*t*_(914)_ = 6.51, *p* < 0.001]. This effect was driven by 22 participants from the German sample who were older than 32 years (age range in the Chinese sample was 18–32 years). Excluding those participants aged above 32 years from the German sample resulted in a mean age of 21.77 (*SD* = 2.84) and no significant difference in age between the two samples [*t*_(892)_ = 1.19, *p* = 0.235]. All analyses within this study were conducted using both the complete German sample and the subsample where participants older than 32 years were excluded. Given there were no substantive differences in the results of these two sets of analyses, all analyses reported below use the complete German sample. In terms of gender, in the Chinese sample there was a higher proportion of male participants than in the German sample [*Chi*^2^_(1)_ = 146.27, *p* < 0.001].

**Table 1:** Descriptive statistics for digital use variables (s-IAT, SAS), IRI empathy, and life satisfaction variables.

	**Chinese sample: *N* = 612 (female, *N* = 162/male, *N* = 450)**			**German sample: *N* = 304 (female, *N* = 207/male, *N* = 97)**		
**Variables**	**Mean**	***SD***	**Skew**	**Mean**	***SD***	**Skew**
s-IAT	31.84 (31.55/31.95)	7.81 (8.17/7.68)	0.12 (−0.02/0.18)	25.18 (24.84/25.92)	6.95 (6.82/7.18)	0.86 (0.67/1.20)
SAS	34.20 (35.47/33.74)	9.30 (9.02/9.37)	−0.30 (−0.35/−0.28)	21.61 (21.96/20.86)	7.45 (7.60/7.09)	0.56 (0.46/0.79)
IRI-PT	15.83 (15.30/16.02)	3.93 (3.79/3.97)	0.04 (0.12/0.00)	17.78 (17.99/17.34)	4.32 (4.44/4.03)	−0.20 (−0.25/0.12)
IRI-FS	15.75 (16.33/15.55)	4.44 (4.33/4.47)	0.04 (00.06/0.08)	18.81 (19.77/16.76)	5.24 (4.79/5.59)	0.49 (0.58/0.16)
IRI-EC	17.72 (18.33/17.50)	3.95 (3.87/3.96)	−0.12 (−0.29/−0.05)	19.60 (20.66/17.34)	4.72 (4.41/4.60)	−0.62 (−0.79/−0.42)
IRI-PD	11.72 (12.54/11.42)	3.85 (3.86/3.80)	−0.05 (−0.12/−0.03)	13.30 (14.36/11.04)	4.38 (4.25/3.76)	0.10 (−0.03/0.27)
LS-Health	7.84 (7.54/7.95)	2.10 (2.13/2.08)	−0.61 (−0.39/−0.69)	7.34 (7.09/7.86)	2.10 (2.26/1.46)	−1.07 (−0.95/−0.63)
LS-Job	6.05 (6.14/5.98)^a^	2.48 (2.54/2.45)^a^	−0.48 (−0.68/−0.34)^a^	7.18 (7.23/7.07)	2.13 (2.09/2.22)	−0.86 (−0.76/−1.03)
LS-Income	5.13 (4.96/5.20)	2.59 (2.69/2.55)	−0.07 (0.12/−0.14)	5.19 (5.08/5.43)	2.74 (2.68/2.86)	−0.13 (−0.06/−0.28)
LS-Housing	7.05 (7.00/7.06)	2.24 (2.05/2.30)	−0.26 (−0.23/−0.27)	7.55 (7.51/7.64)	2.38 (2.41/2.31)	−1.15 (−1.15/−1.15)
LS-Leisure	7.15 (6.96/7.22)	2.36 (2.30/2.38)	−0.39 (−0.40/−0.40)	7.01 (6.97/7.11)	2.08 (2.12/1.99)	−0.93 (−1.08/−0.54)
LS-Family	8.53 (8.22/8.65)	2.21 (2.44/2.12)	−1.05 (−1.11/−0.97)	7.57 (7.61/7.51)	2.30 (2.42/2.03)	−1.08 (−1.17/−0.76)
OLS	7.58 (7.45/7.62)	2.08 (2.00/2.10)	−0.52 (−0.38/−0.56)	7.98 (7.96/8.03)	1.83 (2.01/1.39)	−1.78 (−1.86/−0.75)

s-IAT, short Internet addiction test; SAS, smartphone addiction scale; IRI, interpersonal reactivity index; LS, life satisfaction; PT, Perspective Taking; FS, Fantasy; EC, Empathic Concern; PD, Personal Distress; OLS, overall life satisfaction;

a *in China only N = 88 (female N = 37, male N = 51) provided data for the variable job*.

### Analyses of the questionnaire responses

The questionnaire responses were analyzed for gender effects. In the Chinese sample, no differences were observed for IUD [*t*_(610)_ = 0.56, *p* = 0.577] and SUD [*t*_(610)_ = 1.91, *p* = 0.051]. For empathy, there were no significant differences across gender for PT [*t*_(610)_ = 2.00, *p* = 0.046], FS [*t*_(610)_ = 1.92, *p* = 0.056], and EC [*t*_(610)_ = 2.29, *p* = 0.022] (after Bonferroni adjustment: *p* = 0.05/4 = 0.012). For Personal Distress (PD), a significant difference across gender was found [*t*_(610)_ = 3.19, *p* = 0.001], with female participants having higher scores compared to male participants. No significant differences across gender were found for the life satisfaction variables after applying the Bonferroni adjustment (*p* = 0.05/7 = 0.007). In the German sample, no significant gender differences were found for IUD [*t*_(302)_ = 1.27, *p* = 0.206] and SUD [*t*_(302)_ = 1.20, *p* = 0.230]. In contrast to the Chinese sample, significant differences across gender were found in the German sample for Fantasy (FS) [*t*_(302)_ = 4.83, *p* < 0.001], Empathic Concern (EC) [*t*_(302)_ = 6.04, *p* < 0.001], and Personal Distress (PD) [*t*_(302)_ = 6.58, *p* < 0.001], with females showing higher scores than males for these variables. For PT [*t*_(302)_ = 1.23, *p* = 0.221], no gender difference was found. No significant differences across gender were found for the life satisfaction variables after applying the Bonferroni adjustment (*p* = 0.05/7 = 0.007).

Comparisons across both samples revealed significant differences in IUD [*t*_(866)_ = 12.66, *p* < 0.001] and SUD [*t*_(900)_ = 20.55, *p* < 0.001], with higher scores for both variables in the Chinese sample. Given the gender differences on the empathy variables noted above (e.g., females with higher scores for FS, EC, PD in the German sample), we compared these variables separately for females and males across sample. PT scores differed significantly across sample [*t*_(755)_ = 6.84, *p* < 0.001], as did as those for FS_Female_ [*t*_(367)_ = 7.14, *p* < 0.001], EC_Female_ [*t*_(367)_ = 5.27, *p* < 0.001], and PD_Female_ [*t*_(367)_ = 4.25, *p* < 0.001]. All scores were higher in the German sample. No significant differences were observed across sample for FS_Male_ [*t*_(237)_ = 2.31, *p* = 0.022], EC_Male_ [*t*_(237)_ = 0.35, *p* = 0.726], and PD_Male_ [*t*_(237)_ = 0.90, *p* = 0.3711] scores. Life satisfaction scores in China and Germany differed significantly (Bonferroni adjustment: *p* = 0.05/7 = 0.007) for health [*t*_(810)_ = 3.39, *p* < 0.001], job [*t*_(810)_ = 6.80, *p* < 0.001], housing [*t*_(810)_ = 3.12, *p* = 0.002], family [*t*_(810)_ = 6.11, *p* < 0.001], and overall life satisfaction [*t*_(810)_ = 2.85, *p* = 0.004]. For the health and family variables, higher scores were found in the Chinese sample, whereas the scores for job, housing, and overall life satisfaction were higher in the German sample.

### Associations between IUD/SUD, empathy, and life satisfaction

In both samples robust associations between IUD and empathy were observed; significant positive correlations have been found between the IRI dimension Personal Distress (PD) and IUD in both the Chinese (*r* = 0.32, *p* < 0.001) and the German (*r* = 0.35, *p* < 0.001) samples. For SUD, there were significant positive associations with empathy in the Chinese and German sample, particularly for the IRI-PD variable (China: *r* = 0.32, *p* < 0.001; Germany: *r* = 0.15, *p* = 0.004). Overall, in the German sample, the relationships between empathy and SUD tended to be smaller than those in the Chinese sample and were largely non-significant. The results for both samples are presented in Table 2.

**Table 2:** Partial correlations between digital use variables (s-IAT, SAS) and IRI empathy variables for the complete samples controlling for gender (top line). Correlations are also presented for each variable seperated by gender (female: middle line; male: bottom line).

**Variables**	**s-IAT**	**SAS**	**IRI-PT**	**IRI-FS**	**IRI-EC**	**IRI-PD**
s-IAT	–	0.56^**^	−0.14	0.18^*^	−0.12	0.35^**^
		0.61^**^	–0.21^*^	0.11	−0.19^*^	0.33^**^
		0.47^**^	0.01	0.30^*^	0.04	0.40^**^
SAS	0.60^**^	–	−0.12	0.10	−0.04	0.15^*^
	0.62^**^		−0.14	0.06	−0.10	0.12
	0.60^**^		−0.06	0.17	0.10	0.23
IRI-PT	−0.19^**^	−0.15^**^	–	0.13	0.45^**^	−0.03
	−0.17^*^	−0.11		0.09	0.42^**^	−0.09
	−0.20^**^	−0.17^**^		0.17	0.51^**^	0.02
IRI-FS	0.04	0.11^*^	0.32^**^	–	0.38^**^	0.22^**^
	−0.03	0.07	0.22^*^		0.31^**^	0.08
	0.06	0.12^*^	0.36^**^		0.33^**^	0.24
IRI-EC	−0.15^**^	−0.07	0.35^**^	0.38^**^	–	0.23^**^
	−0.20^**^	−0.12	0.10	0.29^**^		0.07
	−0.14^*^	−0.05	0.45^*^	0.40^**^		0.26^*^
IRI-PD	0.32^**^	0.32^**^	−0.15^**^	0.22^**^	0.03	−
	0.27^**^	0.26^**^	−0.15	0.20^*^	0.09	
	0.34^**^	0.34^**^	−0.13^*^	0.21^**^	0.01	

*s-IAT, short Internet addiction test; SAS, smartphone addiction scale; IRI, interpersonal reactivity index; PT, Perspective Taking; FS, Fantasy; EC, Empathic Concern; PD, Personal Distress. Significant correlations are marked bold*.

* *p < 0.01*,

** *p < 0.001.Correlations for the German sample N = 304; (female, N = 207/male, N = 97) are above the diagonal, correlations for the Chinese sample N = 612; (female, N = 162/male, N = 450) are below the diagonal*.

IUD was negatively associated with life satisfaction in both samples. Negative associations between SUD and life satisfaction were also found, but only in the Chinese sample. For details, please refer to Table 3.

**Table 3:** Partial correlations between digital use variables (s-IAT, SAS) and life satisfaction variables for the complete samples controlling for gender (top line). Correlations are also presented for each variable seperated by gender (female: middle line; male: bottom line).

**Variables**	**SAS**	**LS-Health**	**LS-Job**	**LS-Income**	**LS-Housing**	**LS-Leisure**	**LS-Family**	**OLS**
**CORRELATIONS FOR THE CHINESE SAMPLE *N* = 612; (FEMALE, *N* = 162/MALE, *N* = 450)**								
s-IAT	0.60^**^	−0.16^**^	−0.12^*^^a^	−0.12^**^	−0.11^*^	−0.14^**^	−0.10	−0.18^**^
	0.62^**^	−0.14	−0.28^a^	−0.13	−0.11	−0.12	−0.09	−0.19^*^
	0.60^**^	−0.17^**^	−0.26^a^	−0.11	−0.10	−0.15^*^	−0.10	−0.18^*^
SAS	−	−0.11^*^	−0.07^a^	−0.12^*^	−0.07	−0.11^**^	−0.03	−0.13^**^
		−0.05	−0.29^a^	−0.24^*^	−0.11	−0.14	−0.13	−0.24^*^
		−0.13^*^	−0.04^a^	−0.08	−0.06	−0.10	0.01	−0.10
**CORRELATIONS FOR THE GERMAN SAMPLE *N* = 304; (FEMALE, *N* = 207/MALE, *N* = 97)**								
s-IAT	0.56^**^	−0.13	−0.17^**^	−0.09	−0.13^*^	−0.11	−0.12^*^	−0.15^**^
	0.61^**^	−0.16	−0.18	−0.06	−0.15	−0.13	−0.13	−0.11
	0.47^**^	−0.03	−0.14	−0.14	−0.08	−0.06	−0.11	−0.27^*^
SAS	−	−0.04	−0.12	−0.06	0.01	0.05	−0.02	0.02
		−0.06	−0.11	−0.05	−0.03	0.02	−0.05	0.03
		0.03	−0.12	−0.10	0.11	0.11	0.07	−0.01

s-IAT, short Internet addiction test; SAS, smartphone addiction scale; LS, life satisfaction; OLS, overall life satisfaction;

a *in China only N = 88 (female N = 37, male N = 51) provided data for the variable job. Significant correlations are marked bold*.

* *p < 0.01*,

** *p < 0.001, s-IAT, one sided testing for Germany due to directional hypothesis for IUD*.

Associations between life satisfaction variables and empathy were observed; significant negative correlations have been found between the IRI dimension Personal Distress (PD) and life satisfaction variables in both the complete Chinese (highest correlation for leisure *r* = −0.21, *p* < 0.001) and the complete German (highest correlation for health *r* = −0.21, *p* < 0.001) samples. Again, in the German sample, the relationships between empathy and life satisfaction variables tended to be smaller than those in the Chinese sample. The results for both samples are presented in Table 4.

**Table 4:** Correlations between life satisfaction variables and IRI empathy variables for both samples (top line: complete sample; middle line: female; bottom line: male).

**Variables**	**LS-Health**	**LS-Job**	**LS-Income**	**LS-Housing**	**LS-Leisure**	**LS-Family**	**OLS**
**CORRELATIONS FOR THE CHINESE SAMPLE *N* = 612; (FEMALE, *N* = 162/MALE, *N* = 450)**							
IRI-PT	0.15^**^	0.04^a^	0.09	0.03	0.06	0.07	0.16^**^
	0.07	0.13^a^	0.05	0.15	0.10	0.01	0.07
	0.16^**^	0.02^a^	0.10	−0.01	0.04	0.09	0.19^**^
IRI-FS	−0.09	0.02^a^	0.01	−0.07	−0.08	−0.09	−0.04
	−0.18	0.12^a^	−0.03	−0.10	−0.07	−0.10	−0.05
	−0.05	−0.05^a^	0.02	−0.05	−0.08	−0.08	−0.03
IRI-EC	0.01	0.08^a^	0.01	0.02	0.03	0.10	0.10
	−0.07	0.10^a^	0.02	−0.10	0.02	0.09	0.01
	0.04	0.05^a^	0.02	0.06	0.04	0.12	0.14^**^
IRI-PD	−0.20^**^	0.01^a^	−0.10^*^	−0.18^**^	−0.21^**^	−0.20^**^	−0.21^**^
	−0.28^**^	0.01^a^	−0.16	−0.24^**^	−0.23^**^	−0.28^**^	−0.33^**^
	−0.15^**^	−0.02^a^	**−0.08**	−0.16^**^	−0.20^**^	−0.16^**^	−0.16^**^
**CORRELATIONS FOR THE GERMAN SAMPLE *N* = 304; (FEMALE, *N* = 207/MALE, *N* = 97)**							
IRI-PT	0.08	0.10	0.09	0.06	0.01	0.01	0.01
	0.03	0.07	0.07	0.08	0.09	0.13	0.09
	0.30^*^	0.17	0.16	0.03	0.18	0.02	0.16
IRI-FS	0.01	0.03	−0.05	−0.05	0.08	0.01	0.07
	0.06	0.03	−0.01	−0.07	0.09	0.01	0.10
	0.04	0.01	−0.07	−0.01	0.10	0.02	0.01
IRI-EC	0.07	0.03	0.12	0.12	0.15^*^	0.12	0.17^**^
	0.10	−0.03	0.13	0.14	0.12	0.11	0.18^*^
	0.24^*^	0.13	0.18	0.13	0.27^**^	0.15	**0.22**
IRI-PD	−0.21^**^	−0.16^**^	−0.07	−0.12	−0.10	−0.02	−0.15^*^
	−0.20^**^	−0.18^*^	−0.08	−0.21^**^	−0.20^*^	−0.07	−0.18^*^
	−0.04	−0.20	−0.01	−0.09	−0.18	0.08	−0.04

OLS, overall life satisfaction; IRI, interpersonal reactivity index; PT, Perspective Taking; FS, Fantasy; EC, Empathic Concern; PD, Personal Distress;

a *in China only N = 88 (female N = 37, male N = 51) provided data for the variable job. Significant correlations are marked bold*.

* *p < 0.01*,

** *p < 0.001*.

Fisher's z test was used to test for significant differences of the correlations presented above across both samples (comparing the Chinese and the German sample). The analyses provided no significant results.

**Figure 2:**
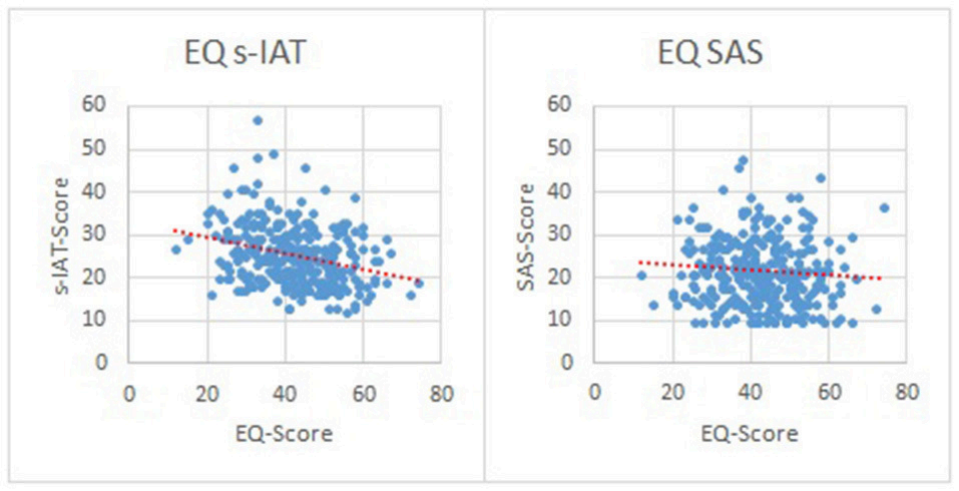
Associations between EQ and IUD/SUD in the German sample (left side: *r* = −0.29, *p* < 0.001; right side: *r* = −0.08, *p* = 0.015).

To demonstrate the robustness of the observed relations between empathy, life satisfaction, and IUD/SUD we also present data measured with the Empathy Quotient Questionnaire; EQ (Baron-Cohen and Wheelwright, 2004). In contrast to the IRI the EQ allows one composite score assessing empathy. Unfortunately, these questionnaire data were only collected in our German sample. Cronbach's α was α = 0.87 in our sample. The following significant correlations were observed: Between EQ and IUD (*r* = −0.29, *p* < 0.001), EQ and SUD (*r* = −0.08, *p* = 0.015), EQ and leisure (*r* = 0.18, *p* < 0.001), EQ and family (*r* = 0.16, *p* = 0.005), EQ and overall life satisfaction (*r* = 0.19, *p* < 0.001). The associations between empathy (EQ) and IUD/SUD are depicted in Figure 2.

### Distribution of IUD and SUD

Using the cut-off values suggested by Pawlikowski et al. (2013) and Kwon et al. (2013), all participants were categorized according to their levels of IUD and SUD. The results revealed a much higher level of IUD and SUD in the Chinese sample compared to the German sample. A summary of these results can be found in Table 5. The mean for IUD in the Chinese sample was *M* = 31.84 (*SD* = 7.81) and in the German sample *M* = 25.18 (*SD* = 6.95). For SUD, the mean in the Chinese sample was *M* = 34.20 (*SD* = 9.30) and in the German sample *M* = 21.61 (*SD* = 7.45). As noted above, these scores from the Chinese sample for IUD and SUD were significantly higher than those from the German sample.

**Table 5:** Distribution of digital use variables (s-IAT, SAS) in percent (Complete sample, female, male).

**Sample**	**s-IAT < 31 (%)**	**s-IAT 31-37 (%)**	**s-IAT > 37**	**SAS ≤ 31/33**	**SAS > 31/33**
**CHINESE SAMPLE *N* = 612; (FEMALE, *N* = 162/MALE, *N* = 450)**					
Total sample	44.6	32.7	22.7	^a^—	^a^—
Female	46.9	30.3	22.8	37.7	62.3
Male	43.8	33.5	22.7	35.1	64.9
**GERMAN SAMPLE *N* = 304; (FEMALE, *N* = 207/MALE, *N* = 97)**					
Total sample	78.9	16.5	4.6	^a^—	^a^—
Female	79.2	16.5	4.3	91.3	8.7
Male	78.4	16.4	5.2	93.8	6.2

s-IAT, short Internet addiction test; SAS, smartphone addiction scale; Cutoff values of s-IAT according to Pawlikowski et al. (2013); cutoff values of SAS according to Kwon et al. (2013): Female = 33, male = 31 (

a *no gender-free cutoff has been provided)*.

## Discussion

The aim of the present study was to extend the existing research on the association between IUD, empathy, and life satisfaction to examine relationships between empathy, life satisfaction and SUD using two samples from diverse cultures. In both samples, earlier findings were partly replicated with regards to the associations between higher IUD and lower empathy, and between higher IUD and lower life satisfaction scores. Higher SUD was significantly associated with higher Personal Distress scores in both the Chinese and German samples. Aside from that, further significant associations between empathy and SUD were only observed in the Chinese sample. Significant negative relationships were found between SUD and life satisfaction, but only in the Chinese sample. Overall, significantly higher mean scores were observed for IUD and SUD in the Chinese sample compared to the German sample.

Our findings confirm earlier evidence showing a negative link between IUD and empathy (Melchers et al., 2015; Jing et al., 2017). As reported in Melchers et al. (2015), more robust associations between the IRI dimensions and IUD were found in the Chinese sample. It should be noted that the associations between FS and PD with IUD are usually positive, whereas the associations between the IRI dimensions EC and PT with IUD are negative (Melchers et al., 2015). In our study, largely the same pattern of associations was detected. Beyond that, we observed a negative association between SUD and empathy, but only for some of the dimensions of the IRI (e.g., higher perspective taking and lower SUD in the Chinese males).

The role of the IRI dimension Personal Distress was particularly noteworthy in this study. We found positive associations between PD and SUD in our Chinese and German samples, indicating that those who are more susceptible to stress in social interactions tend to develop higher SUD. Stress represents a common vulnerability factor for other substance-based addictions also, such as cannabis abuse, indicating that decreased regulation of negative affective states might be a risk factor for the development of addictive patterns of use (Zimmermann et al., 2017). Moreover, one previous study reports results on empathy and SUD (Jeong and Lee, 2015) emphasizing the prominent role of PD for the development of SUD in a sample of nursing students. This is not surprising, because IRI's facet of Personal Distress is strongly linked to neuroticism (Melchers et al., 2016) and neuroticism itself has been often observed to be linked to IUD/SUD (for an overview see Montag and Reuter, 2015; Lachmann et al., 2017a).

Given the results from previous findings that suggest a common personality structure underlying IUD and SUD (Lachmann et al., 2017a), combined with the current findings, it seems plausible to suggest that there is a robust and reliable association between PD and SUD. Nevertheless, it must be clearly noted that not all dimensions of the IRI were associated with IUD or SUD. More specifically, we found robust associations between IUD/SUD and empathy in our Chinese sample, but aside from the link between PD and SUD, we found no significant association between empathy and SUD in our German sample. It needs to be mentioned that the German sample was smaller, hence also the power to detect such an effect was smaller. Moreover, a possible explanation for this finding could be related to the epidemiology of IUD/SUD: Both, IUD/SUD, emerged earlier, and are more currently widespread, in Asian countries (see above). This might be the reason why current data from Asia show a more concerning state of IUD/SUD compared to Europe and, consequently, this could be why we do not yet find a robust association between empathy and SUD in Europe, but more so in Asian countries. It may be that we see associations like this become more prominent in European samples in the future. This point is supported by our findings with regards to the levels of IUD/SUD reported in the Chinese and German samples. Both problematic Internet use and problematic smartphone use were considerably higher in China (IUD: 55.4%, SUD: 63.6%) than in Germany (IUD: 21.1%, SUD: 7.5%). The figures have been calculated using data from Table 5.

We also investigated the association between life satisfaction and IUD/SUD. We supported earlier findings (e.g., Shahnaz and Karim, 2014; Lachmann et al., 2016; Longstreet and Brooks, 2017) by showing a negative association between life satisfaction and IUD in the Chinese and German samples. The findings for relations between life satisfaction and SUD were similar to our results concerning the association between empathy and SUD: all associations were more robust in the Chinese sample. Again, we postulate that the reason for this finding could be the elevated levels of IUD/SUD in the Chinese sample compared to the German sample. We might expect that once a certain threshold of digital addiction has been crossed, a more negative impact on life satisfaction is to be expected.

The comparison of our two samples showed some differences concerning demographic variables: the proportion of males in the Chinese sample was higher compared to the German sample. Furthermore, the mean age in the German sample was higher than in the Chinese sample. This effect was driven by the higher age range in the German sample (18–63 years) compared to the Chinese sample (18–32 years). As outlined in the results section, only 22 participants in the German sample were older than 32 years and these participants caused the significant difference in mean age between the two samples. Since the inclusion or the exclusion of these 22 participants in the analyses had no substantive effect on any of our results, we conducted the analyses with the complete German sample. Nevertheless, because of the observed differences in the proportion of each gender in the samples (even when statistically controlled for), the comparison of means between the two samples must be interpreted with caution. For example, higher values in empathy in the German sample could be caused, at least partially, by the higher number of females in the German sample. In general, females are often described as more empathic than males (Derntl et al., 2010). In this context, it should be noted that gender differences within our two samples were more substantial in the German sample (with females scoring higher on empathy than males). In the Chinese sample these differences were not observed. Even though this might complicate the comparability of empathy scores between the Chinese and German samples, one should keep in mind that a cross-cultural comparison of empathy scores was not our primary goal. The main aim of the present study was to replicate and extend findings concerning the association between IUD/SUD, empathy, and life satisfaction. Given we observed several of the aforementioned associations across two different samples stemming from different cultural backgrounds, the findings from the current study would appear to be relatively robust. It needs to be mentioned that at least for the German sample also EQ data could be presented (see Figure 2). In line with Melchers et al. (2015), a robust negative association between IUD and the EQ could be observed. A similar association could be observed between SUD and the EQ, but at a much lower level. This reflects the aforementioned findings that associations between empathy traits and SUD tend to be lower as with IUD (see also Lachmann et al., 2017a). Unfortunately, we did not assess the EQ data for the Chinese sample, but given the overlap between IRI and the EQ, it is very likely that a similar association would turn up in China. In the earlier work by Melchers et al. (2015) the associations between EQ and IUD was also the most robust pattern to be observed across samples from Germany and China. This likely is the case because the EQ produces a composite score reflecting overall empathy. This also heightens the power to observe general effects between empathy and digital overuse.

This study has some strength and limitations worth noting. Our findings are based on cross-sectional data which makes it impossible to draw conclusions with regards to the causal relationship of the variables included in this study. To achieve this goal further research including experimental and/or longitudinal designs is warranted. Hence, whether low empathy results in IUD/SUD or whether the relationship is the other way around cannot be answered from the present study. Nonetheless, we replicated previous findings and demonstrated several relationships across two independent samples. This shows the robustness of the observed associations. Considering the distribution of gender in our samples, it would have been desirable to have a more balanced distribution of gender within and across the two samples. At least as far as empathy variables in the current study are concerned, the possibility of a gender bias exists because of higher empathy scores, and a higher proportion of females, in the German sample. Findings with regards to empathy should thus be interpreted cautiously.

In sum, the present study confirms earlier findings on the links between IUD, empathy, and life satisfaction and partly extends these findings to SUD. Our findings underline the importance of cross-cultural studies in the area of IUD/SUD, empathy, and life satisfaction. Based on the findings reported here, we suggest that empathy and life satisfaction are potentially important variables in helping us to better understand the etiology and outcomes of IUD and SUD. However, as far as we know, to date no studies have been presented (including the current study) which would allow us to draw causal inferences based on the outlined associations. To work on this topic in future studies would be the next step to develop strategies to protect against the potential overuse of the Internet and smartphones.

## Author contributions

BL, BB, and CM designed the study; CS and RL collected the data for the Chinese sample; BL, CM, RS, and CS collected the data for the German sample; BL performed statistical analyses and wrote the manuscript; CM, BB, BL, RS, CS, AC, and MM critically worked on and refined the manuscript; AC: additionally checked the paper for language use. All authors contributed substantially to the final version of the paper.

### Conflict of interest statement

The authors declare that the research was conducted in the absence of any commercial or financial relationships that could be construed as a potential conflict of interest.
